# Teaching oral and maxillofacial pathology and oral medicine in undergraduate and postgraduate in Latin America

**DOI:** 10.4317/medoral.26773

**Published:** 2025-01-26

**Authors:** Samuel Trezena-Costa, Daniella Reis Barbosa Martelli, Paulo Vitor Pereira Cardoso, Gerardo Gilligan, Janeth Liliam Flores, Wilfredo Alejandro González Arriagada, Claudia Pena Veja, Patricia Reiván Ortiz, Wilson Delgado Azañero, Ronell Bologna Molina, Mariana Villarroel Dorrego, Ricardo Martinez Pedraza, Fábio Ramoa Pires, Bruno Augusto Benevenuto de Andrade, Hercílio Martelli-Júnior

**Affiliations:** 1 Health Science Postgraduate Program, State University of Montes Claros, Montes Claros, Minas Gerais, Brazil; 2 Oral Pathology and Oral Medicine, Dental School, State University of Montes Claros, Montes Claros, Minas Gerais, Brazil; 3 Oral Medicine Department, Faculty of Dentistry, National University of Cordoba, Argentina; 4 School of Dentistry, University of San Andrés, La Paz, Bolivia; 5 Service of Oral and Maxillofacial Pathology, School of Dentistry, University of the Andes, Santiago, Chile; 6 Department of Pathology, National University Hospital of Colombia; Department of Basic Sciences and Oral Medicine, Faculty of Dentistry, National University of Colombia, Colombia; 7 School of Dentistry, Viña del Mar University, Chile; 8 Department of Oral Pathology, Oral Medicine and Oral and Maxillofacial Surgery, School of Dentistry, Universidad Peruana Cayetano Heredia, Lima, Peru; 9 Molecular Pathology Area, School of Dentistry, University of the Republic, Montevideo, Uruguay; 10 Central University of Venezuela, Caracas Venezuela; 11 School of Dentistry, Autonomous University of Nuevo León, Nuevo León, Mexico; 12 Oral Pathology, Dental School, Rio de Janeiro State University, Rio de Janeiro, Brazil; Post-Graduation Program in Dentistry, Estácio de Sá University, Rio de Janeiro, Brazil; 13 Department of Oral Diagnosis and Pathology, School of Dentistry, Universidade Federal do Rio de Janeiro, Brazil; 14 Center for Rehabilitation of Craniofacial Anomalies, Dental School, University of José Rosario Vellano, Alfenas, Minas Gerais, Brazil

## Abstract

**Background:**

Oral and Maxillofacial Pathology (OP) and Oral Medicine (OM) are specialties in dentistry responsible for diagnosis and management of diseases affecting the oral and maxillofacial regions, and to best of our knowledge no information about teaching of OP and OM in undergraduate and postgraduate courses in Latin America have been described. The aim of the present study was to evaluate dimensions related to the teaching of OP and OM in South America and Mexico.

**Material and Methods:**

A structured questionnaire was elaborated and sent to 10 countries, with a professional in each country responsible for answering it. The questions were related to theoretical, laboratory, clinical activities, specialization, residency, master's and PhD courses, and to information from the legislation of OP and OM in each country.

**Results:**

70% of the countries studied, the disciplines of OP and OM are compulsory with workload ranged from 60 to 660 hours. Seven countries have mandatory discipline of OP and 40% have laboratory activities with the study of histopathology. In the case of OM, 60% of the countries have theoretical classes and 40% have mandatory clinical practices. In all ten countries there are laws regulating the opening and operation of undergraduate courses in dentistry. Regarding to the teaching of the postgraduate, the most common was specialization, offered by seven countries, master's degree programs in six countries, while residency is offered by only two countries and the specific PhD in OP/OM offered only in Brazil. Only three countries do not offer postgraduate scholarships and two do not have a research funding agency.

**Conclusions:**

OP and OM are specialties offered at both undergraduate and postgraduate in Latin American countries, with small differences between teaching methodologies in undergraduate and type of training in postgraduate.

** Key words:**Oral Pathology, oral medicine, dentistry, undergraduate, postgraduate.

## Introduction

Oral and Maxillofacial Pathology (OP) and Oral Medicine (OM) are closely related areas of specialization and are considered vital to a comprehensive healthcare system (1). The National Commission on Recognition of Dental Specialties and Certifying Boards of the United States defines OP as the dental specialty that deals with the nature, identification, and management of diseases affecting the oral and maxillofacial regions (https://ncrdscb.ada.org/). OM is considered a “young” specialty of Dentistry. It is recognized around the world as the specialty of Dentistry that interacts with Medicine (2). In Southern Europe and Ibero-American countries, such as Brazil, the term “Stomatology” is used to define the specialty (2).

OM aims to diagnose and to provide (mostly nonsurgical) treatment for primary diseases

of oral mucosa and the jaws, as well as salivary gland disorders, orofacial pain, and maxillofacial manifestations of systemic diseases and their treatment. Some of these conditions include cancer and oral complications of cancer therapy, infectious diseases, and autoimmune disorders, among others (3).

During the COVID-19 pandemic, important proposals and changes were observed in the teaching of the two specialties, both in undergraduate and postgraduate courses and OP services. In a Brazilian study, it was observed significant changes in the attitudes and practices of teaching OP and OM, as an increase in digital platforms and online classes. The lack of students’ interest and the impacts on the professional training need to be considered as a factor that may reflect on clinical practice. The attention on difficulties to access the internet highlight the need to provide digital inclusion policies (4). In Canada and the U.S., about 89% oral pathologists were able to provide emergency care, with 82.4% and 23.5% having performed telehealth consultations and oral biopsy procedures, respectively (5).

Another study showed that adaptation in laboratory workflow, the advent of digital pathology and remote reporting can mitigate the impact of similar future disruptions to the oral and maxillofacial pathology laboratory workflow avoiding delays in diagnosis and report, to facilitate timely management of head and neck cancer patients (6). We recently evaluated remote teaching in postgraduate programs (master's). Despite the difficulties, remote teaching proved to be an effective alternative to face-to-face teaching (7).

Some studies around the world have analyzed the state of art and professional perspectives in OP and OM (1,5,8-11). However, no previous study has evaluated the teaching of OP and OM in undergraduate and postgraduate courses in Latin America. Analyzing the data in question will be useful in identifying gaps regarding OP and MO teaching in Latin America. In addition, identifying negative and positive points will be essential in the standardization of a scenario with strong potential in OP and MO. Thus, the aim of the present study was to evaluate dimensions related to the teaching of OP and OM in Latin America.

Materials and Methods

- Participants

A cross-sectional, descriptive, and convenience study was conducted. Ten Latin America countries participated in the study (Argentina, Bolivia, Brazil, Chile, Colombia, Ecuador, Peru, Uruguay, Venezuela and Mexico). In each country, a professional responsible for collecting the requested data was chosen. All professionals involved have training in the areas of OP/OM and work in universities.

- Data collection

A structured questionnaire was elaborated, with two major dimensions, undergraduate and postgraduate teaching. In undergraduate and postgraduate teaching, the questions were related to OP and OM. After the invitation of the OP/ OM specialists, we sent a structured questionnaire, by personal e-mail, involving undergraduate and postgraduate teaching from each country. The information described by the collaborators was related to laws and regulatory technical notes of each country, for private and public schools. Data were extracted from government or open access websites. The questionnaires were sent at the beginning of December 2023, with a deadline for data collection until the end of January 2024. After receiving all the questionnaires, the experts were again consulted for clarification of doubts and standardization of information. All participating countries answered the questionnaire and were included in the study. In the dimension of undergraduate teaching, the questions were related to theoretical, laboratory and clinical activities and to information from the legislation of each country on the disciplines of OP and OM. In the dimension of postgraduate teaching, the questions were related to specialization, residency, master's and PhD courses, as well as the legislation and offer of scholarships to be carried out in each country.

- Statistical analysis

When questionnaires returned, the data were consolidated into Microsoft Excel® spreadsheets for descriptive analysis of the information. The study used public and secondary data, not requiring submission to the Research Ethics Committee.

## Results

The questionnaires were returned from all countries participating in the study. Table 1 shows the general data in relation to the quantitative dimensions of OP and OM in the curriculum of undergraduate courses in the ten countries in Latin America. The average duration of undergraduate courses ranged from 4 to 6 years. However, the most common was the duration of 5 years, verified in 6 countries of the study. In all countries, the undergraduate course is face-to-face, and the modality is more common full time. In general, the total workload of Dentistry courses ranged from 4,000 (Brazil) to 8,640 hours (Colombia). In 70% of the countries studied, the disciplines of OP and OM are compulsory.

Only in Brazil, Chile and Peru these disciplines are not compulsory. However, although OP and OM are not mandatory disciplines according to the curriculum regulations of these countries, they are present in all courses and universities in Chile. In Brazil case, OP and OM (designated as Stomatology) in the curriculum is not mandatory, but there must be teaching during graduation in all institutions. In Peru it is only mandatory at "national" universities. The workload of the disciplines of OP and OM ranged from 60 to 660 hours.

Of the countries that have mandatory laws requiring the presence of OP/OM disciplines, all include theoretical classes about OP. However, only four of these countries (Argentina, Colombia, Mexico, and Uruguay) have laboratory activities involving the study of histopathology. Of these seven countries, only Bolivia does not have theoretical and clinical classes of OM. Oral Medicine clinical classes are present in Argentina, Colombia, Uruguay, and Venezuela. Although there is no mandatory requirement for these disciplines in Brazil, Chile, and Peru, all courses include theoretical classes in OP. In these three countries, the presence of laboratory classes in OP, theoretical classes and clinical classes in OM varies among educational institutions. However, in Brazil and Chile, the theoretical content of OM must be taught to undergraduates, although clinical practice for public service is not available in all institutions.

In all ten countries there are laws regulating the opening and operation of undergraduate courses in dentistry. In Brazil, Mexico, and Venezuela, this role is carried out by only one Ministery, namely the Ministry of Education, the Ministry of Public Education, and the Ministry of Higher Education respectively. In the other countries, there are additional agencies, departments, and commissions that are responsible for accrediting new dentistry courses. Mostly, these are public organs that assist the Ministries of Education of each country in supervising these courses. In the case of the Chile, private universities have their own autonomy to open courses; however, they still require approval and supervision from public entities. In Bolivia, Public Universities have autonomous character and are financed by the Bolivian State, while Private Universities are controlled and depend on the Ministry of Education.Only in Brazil there is government programs aimed at assisting the admission to undergraduate courses in private institutions. The University for All Program (Prouni in Portuguese) and the Student Financing Fund (FIES in Portuguese) offer full and partial scholarships, as well as provide financing to students in non-free higher education courses.

Regarding to the teaching of the postgraduate (specialization, residency, master's and PhD) in OP/OM, Table 2 shows the duration of each of the existing modalities of postgraduate courses in Latin America country, adapted from Santos-Leite *et al*., (12), The most common was specialization, offered by seven countries with varying durations from 1.5 to 3 years. The most unusual modalities were residency, offered by only two countries (Brazil and Peru) and the specific PhD in OP/OM offered only in Brazil. Master's degree programs in six countries (Argentina, Brazil, Mexico, Peru, Uruguay and Venezuela) and have a duration of 2 to 3 years.

Table 3 shows the reality of the ten countries studied in relation to opening and supervision of postgraduate courses, existence of scholarships and research funding agencies. In all countries there is an official Institution for the opening of postgraduate courses, however, in the case of monitoring and supervision, only Venezuela does not have. Only three countries do not offer postgraduate scholarships (Bolivia, Uruguay and Venezuela) and Bolivia and Venezuela do not have a research funding agency.

## Discussion

Recently, we conducted a study evaluating the quantitative distribution of OP and OM professionals, as well as professional training and fields of work in the countries of Latin America. We note that, although both specialties are globally practiced, there is a disparity in the training of these professionals in the countries studied (12). To the best of our knowledge, the present study was the first to evaluate quantitative data on the teaching of OP and OM in undergraduate and postgraduate courses in Latin America. In general, the data on the curricula of the undergraduate courses were very general, as well as the teaching of OP and OM.

A study evaluating the teaching of OM in the United Kingdom and Ireland showed that the curriculum will act as a foundation for an increasingly shared approach between Centers with respect to the outcomes to be achieved in OM (13). It is observed that there is no consensus and alignment of the teaching of OM in European countries (14,15), which we also observed in the ten Latin America countries studied here, including the teaching of OP. The workloads of the disciplines of OP and OM varied widely (60 to 660 hours). One piece of information that was not analyzed in our study, but would be important to know, is the content of OP and OM curricula in the different Latin America countries. Recently, a study evaluated the teaching of oral cancer and potentially malignant lesions in dental schools in Mexico (16). Frailties in teaching regarding potentially malignant lesions and oral cancer were identified in some Mexican institutions. The study concludes that curricular integration and standardization among other countries are necessary to improve the performance of OP/OM (16).

In relation to theoretical, laboratory and clinical classes, it was observed that the most common activities among the countries studied were the theoretical classes of OP and OM. When analyzing OP laboratory activities and OM clinics, there is a variation between the countries studied. In the case of OP laboratory classes, in three countries they are not offered, in one it is not required, and in Peru, it varies between institutions, situation similar to the clinical activities of OM. An international study (10) proposed a competency list that, although not intended to be prescriptive or reductionist, could provide a model for countries developing an advanced training curriculum for OM. More competencies could be added, depending on the local health care needs and regulations. In Brazil, it is verified that a significant number of dental schools have theoretical classes, laboratory and clinical activities, but according to the National Curriculum Guidelines of the Ministry of Education, these disciplines are not required. The current Brazilian legislation describes the general and specific competencies that a dental student must receive during the undergraduate course (http://portal.mec.gov.br/docman/junho-2021-pdf/191741-rces003-21/file). Even not presenting disciplines called OP and OM, the content should be provided for training dentists.

During the COVID-19 pandemic, different methods of teaching OP were observed, which has contributed to reflections and important changes observed after the end of the pandemic. Regarding satisfaction with remote teaching, the undergraduates reported that they liked to study independently and were participative in classes. In contrast, the advisors reported good adaptation and motivation for remote teaching. In fact, remote teaching has advantages such as low cost, flexible schedules and convenience which can provide greater ease of adaptation to teaching (17). In addition, adaptations were made to laboratory routines, and remote histopathology was introduced, allowing the continuity of academic training (6).

A study conducted with postgraduate students from OP and OM Programs in Brazil showed that the adoption of remote teaching proved to be effective during this period (7). However, it is seen that some in-person practical activities that have been suspended are essential for training. The adoption of teleconsulting by educational institutions, during the pandemic was a fundamental strategy for continuing care and maintaining teaching about the management of oral diseases (18).

Our study evaluated countries with very different populations and socio-economic conditions. The most common type of training among OP and OM practitioners was a specialization course and a master's degree, found in seven and six countries, respectively. The duration of the specialization courses ranged from 1.5 to 3 years, while the master's degree course most commonly lasts 2 years. Currently we have only one specific PhD course in OP and OM in the countries studied in Latin America. It is observed that training in OP and OM is quite variable worldwide. A study carried out in the area of OP involving 42 countries affiliated to IAOP showed that there is enough common ground for the development of an agreed indicative framework about education and training in OP (9). Bez *et al*. (19) pointed out that despite a broad agreement in OM syllabi and clinical practice; there are still some important differences in its definition and scopes within Europe. It is crucial that European countries agree a consensus definition of OM and clarify competencies and limits, so they may move from institution and region-specific approaches to an international framework. According to the European Directives, it is timely to recognize a minimum three year standard curriculum at a postgraduate level which will lead to uniformity of training for OM residencies in European country members and will eventually provide guidelines for a broader OM specialty recognition. These considerations are essential for the greater and better development of OP and OM. This scenario also applies to Latin America.

The performance of research in the master's and PhD programs, as well as the granting of scholarships are essential for the consolidation and advancement in any area of knowledge. There is a direct correlation between scientific production and economic development, including the biomedical area (20). Latin America is lacking behind in biomedical development, and it needs to generate important knowledge specific to the region and to increase its contribution to the global research community (21). In recent years, particularly in the areas of OP and OM, we have observed important indicators of growth in scientific production (22) and internationalization of Brazilian researchers (23), but these facts cannot be discontinued due to varied economic Investments (24).

Some limitations of the study were the lack of knowledge of the subjects developed in the areas of OP and OM during the undergraduate course, as well as the lines of research developed in the postgraduate programs. It was also not possible to know the amount of investment in scholarships and funding for research projects in the countries studied.

In summary, our study showed that the disciplines of OP and OM are offered in virtually all countries studied. The postgraduate programs have similar durations and only Brazil offers a specific PhD course in the specialties studied. We believe that studies in this line of research will allow us to deepen the knowledge and expand the discussions of the teaching of OP and OM in Latin America.

## Figures and Tables

**Table 1:** Oral Pathology (OP) and Oral Medicine (OM) data in undergraduate Dentistry courses in Latin America.

Country	Dentistry course' duration (years)	Curriculum modality	Course workload (hours)	Mandatory OP and OM in the curriculum	OP/OM workload (hours)
Argentina	5	First year partial. Last years full time	4,500	Yes	120 to144
Bolivia	5	First year partial. Last years full time	6,300 to 7,300	Yes	436
Brazil	4 to 5	Some are partial and other full time	Minimum 4,000	No*	-
Chile	5,5 to 6	Full time	Minimum 5,000	No**	120-480
Colombia	5	Full time	8,640	Yes	240
Ecuador	5	Full time	8,000	Yes	105
Mexico	4 to 6	Partial time	4,779 to 7,900	Yes	80 to 100
Peru	5	Full time	5,195	No***	60 to 480
Uruguay	5,5	Full time	7,425	Yes	660
Venezuela	5	Full time	Not specified	Yes	480

*In accordance with the new curricular guidelines for dentistry courses, it is not mandatory to include disciplines specifically designated as Oral Pathology/Oral Medicine. However, the content must be taught during the undergraduate program. ** There are no laws requiring the presence of the discipline, but it is included in all undergraduate courses. ***Mandatory only at national universities.

**Table 2:** Duration of specialization, residency, master's and PhD courses in Oral Pathology/Oral Medicine in Latin America.

Country	Specialization	Residence	Master Degree	PhD Degree
	Duration (Years)	Duration (Years)	Duration (Years)	Duration (Years)
Argentina	2 to 3	NA	3	NA
Bolivia	2	NA	NA	NA
Brazil	1,5	2	2	4
Chile	2	NA	NA	NA
Colombia	2	NA	NA	NA
Ecuador	NA	NA	NA	NA
Mexico	2	NA	2	NA
Peru	3	3	2	NA
Uruguay	NA	NA	2	NA
Venezuela	NA	NA	2	NA

*NA=Not applicable.

**Table 3:** Opening and supervision of postgraduate courses, presence of scholarships and research funding agencies in Latin America.

Country	Opening of postgraduate courses	Supervision of postgraduate courses	Postgraduate scholarships	Research funding agencies
Argentina	Ministry of Education of the Argentine Nation		PhD	Yes
Bolivia	Executive Committee of the Bolivian University	Department of Research, Postgraduate Studies and Social Interaction	No	No
Brazil	Specialization: Ministry of Education; Residence: Council Class and Ministry of Health; Master and PhD: Foundation Coordination for the Improvement of Higher Education Personnel	Foundation Coordination for the Improvement of Higher Education Personnel	Yes	Foundation Coordination for the Improvement of Higher Education Personnel, Ministry of Health and National Council for Scientific and Technological Development, and State Research Support Foundations
Chile	Autonomy of Universities subject to approval by the Ministry of Education	National Accreditation Commission	Yes	Yes National Research and Development Agency
Colombia	Ministry of Education		Yes*	Yes Ministry of Health, Education and Sciences
Ecuador	The Organic Law on Higher Education and the Higher Education Council	Higher Education Council	Yes	Yes Secretariat of Higher Education, Science, Technology and Innovation. In conjunction with Universities through research councils and international donor agencies
Mexico	Public Education Department	Academic organs and curriculum committees of each institution or University	Yes	Yes National Council of Science and Technology
Peru	Ministry of Education and National Superintendency of University Higher Education	Each University has a corresponding entity	Yes	Yes National Council of Science and Technology and Hipolito Unanue Institute
Uruguay	Postgraduate Academic Committee		No	Yes Scientific Research Sector Commission and Agency for Research and Innovation
Venezuela	Ministry of Higher Education	There are no responsible organs	No	No

*Scholarships offered only at universities that have their own financing programs.
